# Thrombopoietin knock-in augments platelet generation from human embryonic stem cells

**DOI:** 10.1186/s13287-018-0926-x

**Published:** 2018-07-17

**Authors:** Leisheng Zhang, Cuicui Liu, Hongtao Wang, Dan Wu, Pei Su, Mengge Wang, Jiaojiao Guo, Shixuan Zhao, Shuxu Dong, Wen Zhou, Cameron Arakaki, Xiaobing Zhang, Jiaxi Zhou

**Affiliations:** 1 State Key Laboratory of Experimental Hematology, Institute of Hematology & Blood Diseases Hospital, Chinese Academy of Medical Sciences & Peking Union Medical College, 288 Nanjing Road, Tianjin, 300020 China; 2 Center for Stem Cell Medicine, Chinese Academy of Medical Sciences & Department of Stem Cells and Regenerative Medicine, Peking Union Medical College, Beijing, China; 3 School of Basic Medical Science and Cancer Research Institute, Central South University, Changsha, 410013 China; 4 Division of Regenerative Medicine MC 1528B, Department of Medicine, Loma Linda University, 11234 Anderson Street, Loma Linda, CA 92350 USA

**Keywords:** Thrombopoietin, Knock-in, Early hematopoiesis, Platelets, Human embryonic stem cells

## Abstract

**Background:**

Refinement of therapeutic-scale platelet production in vitro will provide a new source for transfusion in patients undergoing chemotherapy or radiotherapy. However, procedures for cost-effective and scalable platelet generation remain to be established.

**Methods:**

In this study, we established human embryonic stem cell (hESC) lines containing knock-in of thrombopoietin (TPO) via CRISPR/Cas9-mediated genome editing. The expression and secretion of TPO was detected by western blotting and enzyme-linked immunosorbent assay. Then, we tested the potency for hematopoietic differentiation by coculturing the cells with mAGM-S3 cells and measured the generation of CD43^+^ and CD45^+^ hematopoietic progenitor cells (HPCs). The potency for megakaryocytic differentiation and platelet generation of TPO knock-in hESCs were further detected by measuring the expression of CD41a and CD42b. The morphology and function of platelets were analyzed with electronic microscopy and aggregation assay.

**Results:**

The TPO gene was successfully inserted into the AAVS1 locus of the hESC genome and two cell lines with stable TPO expression and secretion were established. TPO knock-in exerts minimal effects on pluripotency but enhances early hematopoiesis and generation of more HPCs. More importantly, upon its knock-in, TPO augments megakaryocytic differentiation and platelet generation. In addition, the platelets derived from hESCs in vitro are functionally and morphologically comparable to those found in peripheral blood. Furthermore, TPO knock-in can partially replace the large quantities of extrinsic TPO necessary for megakaryocytic differentiation and platelet generation.

**Conclusions:**

Our results demonstrate that autonomous production of cytokines in hESCs may become a powerful approach for cost-effective and large-scale platelet generation in translational medicine.

**Electronic supplementary material:**

The online version of this article (10.1186/s13287-018-0926-x) contains supplementary material, which is available to authorized users.

## Background

Platelets, produced by megakaryocytes (MKs) (2 × 10^3^–10 × 10^3^ platelets per megakaryocyte) [1], are an essential blood constituent (15× 10^10^–45 × 10^10^/L) and function critically in hemostasis, wound healing, immunity, inflammation, and thrombocytopenia therapy [1, 2]. Moreover, platelets are anucleate and can be irradiated before transfusion, making them safer than other blood cell fractions [3, 4]. However, due to their short life span (3–7 days), their need for room-temperature storage, and limitations of HLA-ABC transplanting typing, the donor-supplied platelets are insufficient to meet the current transfusion needs of patients [2, 5].

Human pluripotent stem cells (hESCs), including human embryonic stem cells (hESCs) and human induced pluripotent stem cells (hiPSCs), have the ability for long-term self-renewal and the capacity for multilineage differentiation [6, 7], are amenable to genetic manipulations, and have been demonstrated as an excellent alternative to generate donor-independent platelets for clinical transfusion and a unique model for biological study of human megakaryopoiesis and thrombopoiesis [8, 9]. For example, Kaufman et al. [10] first reported the generation of megakaryocyte colonies from hESCs, while Gaur et al. [11] established an OP9 stromal cell coculture system to generate megakaryocytes from hESCs. In addition, Takayama et al. [4] obtained functional platelets from hESCs in vitro via embryonic stem cell sacs, VEGF-promoted structures, for the first time. More recently, multiple cytokines, chemical molecules, and transcription factors have been tested for development of large-scale platelet production [2, 5, 8, 12]. Lu et al. [1] and Feng et al. [2] efficiently induced hESCs and hiPSCs into platelet-producing megakaryocytes with cytokine and small molecule combinations. A “forward programming” (FOP) strategy has also been used to successfully generate megakaryocytes via overexpression of *GATA1*-*FLI1*-*TAL1* in hESCs. To derive platelets on a large scale, Nakamura et al. [5] successfully established immortalized megakaryocyte progenitor cell lines (imMKCLs) from hESC-derived hematopoietic progenitors via *BMI1*, *BCL-XL*, and *c-MYC* overexpression. We have recently reported the use of a three-dimensional (3D) rotary culture system integrated with biophysical and biochemical signals resulting in significantly augmented megakaryopoiesis and thrombopoiesis. All of these studies have demonstrated that functionally intact platelets are capable of being generated on a large scale from hESCs. However, the current strategies are inefficient and rely heavily on the addition of a variety of high-dose cytokines, thus making them unfeasible to produce affordable quantities of platelets from hESCs for transfusion and therapeutic purposes. Thus, replacing expensive cytokines with chemical compounds and/or autonomous production of cytokines by engineered hESCs might facilitate large-scale platelet production from hESCs for clinical purposes.

Thrombopoietin (TPO) is the primary regulator of megakaryopoiesis and platelet production [13] and is currently regarded as a principal panhematopoietic cytokine [14]. The dysregulation of TPO/c-MPL expression leads to hematological disorders, and c-MPL agonists or TPO mimics have been shown to be effective treatments in patients with thrombocytopenia [15, 16]. TPO^−/−^ or c-MPL^−/−^ mice develop normally but show deficiencies in megakaryocytes and are severely thrombocytopenic [17, 18], while mice intraperitoneally injected with recombinant TPO protein increase their circulating platelet levels by more than 4-fold [19]. Based on these in-vitro and in-vivo studies, we generated TPO knock-in hESCs and tested their potential in megakaryopoiesis and thrombopoiesis. Our results demonstrate that TPO knock-in in hESCs enhances early hematopoietic differentiation, megakaryocyte generation, and platelet derivation. More importantly, our results provide the first proof-of-concept study to show that knock-in of extrinsic cytokines in hESCs can replace, at least partially, the exogenous source and thus might significantly reduce the cost for large-scale platelet generation from hESCs for future transfusion purposes.

## Methods

### Maintenance of human embryonic stem cells

H1 hESCs (WiCell Research Institute) were cultured in mTeSR (Stem Cell Technology) and seeded on Matrigel (BD Biosciences)-coated tissue culture plates (Thermo Fisher Scientific) in 37 °C, 5% CO_2_ incubators. H1 hESCs were passaged every 3 days with the use of 2 U/ml dispase (StemCell Tech) at a dilution of 1:5, according to the manufacturer’s instructions. For more details, see Additional file 1: Additional Experimental Procedures.

### Construction of the targeting plasmid

The CHOPCHOP website (https://chopchop.rc.fas.harvard.edu/) was utilized to design a high-performance sgRNA targeting the human AAVS1 locus. Based on our prior experience, we preferentially chose a sgRNA with a G at the 5′ end which initiates U6-promoter-mediated transcription [20]. The sgRNA used in this study was sgAAVS1b.

Both the Cas9 and sgRNA plasmids were constructed with a NEBuilder HiFi DNA Assembly Kit (New England Biolabs). Multiple colonies were chosen for Sanger sequencing (MCLAB) to identify the correct clones using the primers U6-F, EF1-F, and wpre-R. Correct clones were grown in CircleGrow Media (MP Biomedicals) and DNA plasmids were purified using Endo-Free Plasmid Maxi Kits (QiaGen).

The TPO-E2A-GFP and GFP donor plasmids used in this study were generated with a CloneJET PCR Cloning Kit (Thermo Scientific). Multiple colonies were chosen for Sanger sequencing (MCLAB) to identify the correct clones using the primers pJET1.2-F and pJET1.2-R. Correct clones were cultured, and DNA plasmids were purified, as described previously [20] (See also Additional file 1: Additional Information for details). All of the primer sequences are listed in Additional file 1: Table S1.

### Establishment of TPO knock-in H1 hESCs

H1 hESCs were cultured in mTeSR supplemented with 25 μg/ml plasmocin™ (InvivoGen) for 2 weeks and pretreated with 5 μM ROCK inhibitor Y27632 1 day prior to transfection. Next, donor vectors were delivered using the Amaxa™ human stem cell Nucleofector™ kit 2 (Lonza), according to the manufacturer’s instructions. Briefly, 1 × 10^6^ single H1 hESCs were collected and resuspended in transfer buffer (82 μl Nucleofector solution and 18 μl supplement) mixed with a cocktail of 3 μg 717-pEF1-Cas9-wpre-polyA, 3 μg S479-pU6-AAVS1b vector, and 6 μg pD-AAVS1 (HA600)-EF1α-TPO-GFP or 6 μg pD-AAVS1 (HA600)-EF1α-GFP. Electroporation was performed using the “B-16” protocol of the Nucleofector™ 2b Device (Lonza). At 4 h and 12 h after electroporation, fresh mTeSR supplemented with 5 μM ROCK inhibitor Y27632 was added. At 24 h after electroporation, the medium was changed to fresh mTeSR without ROCK inhibitor Y27632 and was subsequently changed daily. After three passages, the GFP-positive H1 hESCs were enriched with flow cytometry-based sorting. To establish the GFP and TPO-GFP knock-in H1 hESC stable lines, 2 × 10^4^ single cells were seeded on Matrigel (BD Biosciences)-coated six-well plates (Corning). Around 3– 5 days later, single clones expressing bright green fluorescence were selected for expansion and detection as reported previously [21].

### Real-time PCR

Cells at different time points were collected and dissociated with TRIZOL reagent (Invitrogen). Total mRNAs were extracted, according to the manufacturer’s instructions. cDNA was produced using a reverse transcription kit (Promega). The expression of *ACTIN*, *GATA1*,* FLI-1*, *RUNX1*,* ITGB3*, and *NF-E2* genes was detected using a 7900HT Fast Real-Time PCR system (7900HT; ABI) with the QuantiTech SYBR Green PCR kit (BioRad). The primers for gene detection are presented in Additional file 1: Table S1.

### Measurement of TPO expression

The secretory TPO protein in the supernatant was collected by centrifugation at 600 × *g* for 30 min and enriched using a protein purification kit (Millipore). The expression of TPO was quantified using western blotting (detailed information regarding antibodies is presented in Additional file 1: Table S2.) or a Human TPO enzyme-linked immunosorbent assay (ELISA) kit (Neobioscience), according to the manufacturer’s instructions.

### Teratoma assay

The teratoma formation assay was performed as reported previously [22] in accordance with internationally recognized guidelines. Briefly, 4-week-old NOD/SCID mice were provided by and approved (approval no. KT2016011-EC-1) for use by the Peking Union Medical College Institutional Animal Care and Use Committee (license no. SCXK & SYXK 2005-0001, Tianjin) and maintained under specific pathogen-free conditions. Approximately 1 × 10^7^ TPO-GFP or GFP knock-in H1 hESCs were harvested and suspended within Matrigel (Corning) for intramuscular injection into the hind leg. At 6 weeks after injection, euthanasia was performed and teratomas were dissected. The morphology of teratomas was visualized under a live imager (IVIS Lumina II). Tissue histologic section and hematoxylin–eosin (HE) staining were performed by the Department of Pathology, Institute of Hematology & Blood Diseases Hospital, Chinese Academy of Medical Sciences & Peking Union Medical College.

### Hematopoietic differentiation of hESCs

Hematopoietic differentiation of hESCs with the mouse AGM-S3 cell coculture system were performed as described previously [23]. Briefly, H1 hESCs were dissociated into single cells and seeded at a density of 5 × 10^4^ cells/ml in mTeSR supplemented with ROCK inhibitor Y27632 (10 μM) on Matrigel-coated tissue culture plates (Thermo Fisher Scientific). After 48 h, the colonies were dissociated into small colonies with 2 U/ml dispase (Roche), and seeded onto mAGM-S3 stromal cells in mTeSR at a density of 5–7 colonies/ml. After 24 h, mTeSR was removed, and hESC early hematopoietic differentiation medium (see also Additional file 1: Additional Information for details) was added and subsequently changed daily.

### Colony-forming unit assay

At day 12 of hematopoietic differentiation, cobblestone-like HPCs were mechanically isolated as described previously [23]. Briefly, 2 × 10^4^ HPCs were seeded into methyl cellulose semi-solid culture medium (MethoCult H4435; StemCell Tech), according to the manufacturer’s instructions. After 2 weeks, colony-forming units (CFUs) were counted.

### Megakaryocytic differentiation and platelet generation from HPCs

Megakaryocyte differentiation and platelet generation were practiced as reported previously [23]. Briefly, HPCs at day 12 of hematopoietic differentiation were collected and seeded at a density of 1 × 10^5^ cells/ml in hematopoietic differentiation media, supplemented with SCF (20 ng/ml), IL-3 (10 ng/ml), IL-6 (10 ng/ml), IL-9 (10 ng/ml), IL-11 (10 ng/ml), ROCK inhibitor Y27632 (10 μM), and TPO at different concentrations (0, 10, 20, 50 ng/ml) according to the experiment design. The media were changed every 3 days. Detailed information regarding chemical compounds is presented in Additional file 1: Table S3.

### Flow cytometry

HPCs at different time points of differentiation were collected, washed with 1× PBS twice, incubated with APC-CD43 (BD Biosciences) or PE-CD45 (BD Biosciences), and analyzed with a BD FACSCanto II (BD Biosciences) flow cytometer. Megakaryocytes were collected and labeled with APC-CD41a and PE-CD42b antibodies. Platelets in the supernatant were enriched and labeled with APC-CD41a, PE-CD42b, or PE-CD62p antibodies and detected with a BD FACS Aria II (BD Biosciences) flow cytometer as reported previously [24]. Detailed information regarding antibodies is presented in Additional file 1: Table S2.

### Ploidy analysis and electron microscopy of megakaryocytes

Ploidy analysis of megakaryocytes was performed as reported previously [24]. For ploidy analysis, cells were labeled with CD41a antibody (BD Biosciences) and propidium iodide (Sigma-Aldrich), and subsequently analyzed by gating the CD41a^+^ population using a BD FACSCanto II (BD Biosciences) flow cytometer. For electron microscopy analysis, megakaryocytes were analyzed as reported previously [24].

### Enrichment and immunofluorescence of platelets

Platelet enrichment and purification were performed as reported previously [24]. The purified platelets were resuspended in CGS buffer and maintained at room temperature for further morphological and functional assays. For immunofluorescence assay, platelets were plated onto dishes or coverslips (Corning) coated with 3poly-L-lysine (Beyotime). After fixation and permeabilization, platelets were labeled with different antibodies. Fluorescent images of platelets were recorded and analyzed as described previously [24].

### Aggregation and adhesion test of platelets

The aggregation and adhesion potential of platelets was evaluated as reported previously [24]. For aggregation potential analysis, the Calcein-AM (Invitrogen)-labeled platelets were mixed with mouse anti-β1-tubulin (GE Healthcare)-labeled peripheral blood platelets. After incubation with agonists, the aggregates were labeled with 594-conjugated goat anti-mouse IgG (Bio). For adhesion potential assay, platelets were incubated with or without thrombin (Sigma) and then labeled with phalloidin (Invitrogen). Images of platelets were visualized under a confocal microscopy (LSM710).

### Statistical analysis

All data were shown as mean ± SEM (*n* = 3, representing three independent experiments). Statistical calculations were performed using GraphPad Prism 5 software (version v5.01). Differences were considered statistically significant when *P* < 0.05.

## Results

### Establishment of TPO knock-in hESC lines

**Fig. 1:**
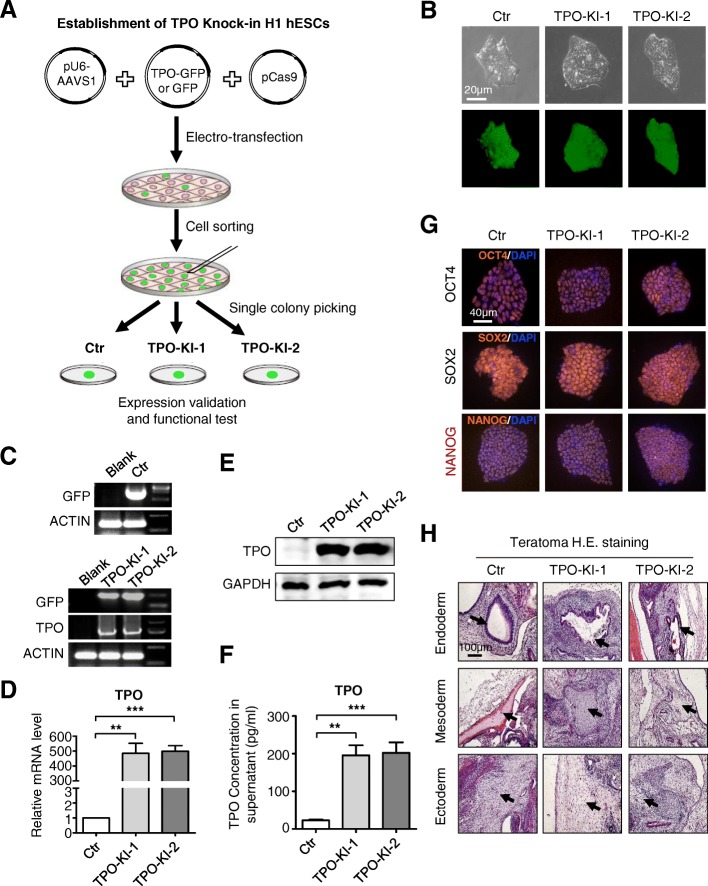
Establishment of TPO knock-in hESC lines. **a** Schematic of TPO-KI stable cell line establishment. GFP used as control (Ctr). **b** Phase-contrast images (top panel) and fluorescence images (bottom panel) of Ctr or TPO-KI-1/-2 H1 hESCs in mTeSR. Scale bar = 20 μm. **c** Identification of Ctr or TPO-KI-1/-2 H1 hESCs using agarose gel electrophoresis of amplified PCR products with designed primers listed in Additional file 1: Table S1. **d** qRT-PCR analysis of TPO expression in Ctr or TPO-KI-1/-2 H1 hESCs in mTeSR. All values normalized to level (=1) of mRNA in Ctr. Data shown as mean ± SEM (*n* = 3). **P < 0.01; ***P < 0.001. **e** Western blotting analysis confirms expression of TPO in Ctr or TPO-KI-1/-2 H1 hESCs in mTeSR. GAPDH used as loading control. **f** ELISA shows soluble TPO in supernatant of Ctr or TPO-KI-1/-2 H1 hESCs after 48 h culture in mTeSR. All values normalized to level (=1) of TPO concentration in Ctr. Data shown as mean ± SEM (*n* = 3). **P < 0.01; ***P < 0.001. **g** Immunofluorescence images of pluripotency markers (POU5F1, SOX2, NANOG) in Ctr or TPO-KI-1/-2 H1 hESCs in mTeSR. Scale bar = 40 μm. **h** Hematoxylin and eosin (H.E.) staining of representative structures in teratomas formed by Ctr or TPO-KI-1/-2 H1 hESCs. Scale bar = 100 μm. DAPI 4′,6-diamidino-2-phenylindole, GAPDH glyceraldehyde 3-phosphate dehydrogenase, GFP green fluorescent protein, hESC human embryonic stem cell, TPO thrombopoietin

To generate large-scale production of platelets in vitro for transfusion, we recently described a 3D rotary culture system integrated with biophysical and biochemical signals to enhance the efficiency and function of platelet generation [24]. Despite the advantages of this strategy, high concentrations of TPO (50 ng/ml) and other cytokines are still necessary in the culture medium, making it costly for large-scale generation of platelets. Thus, we aimed to integrate TPO and generate stable cell lines for autonomous TPO expression in the attempt to replace the need for an exogenous source. We therefore constructed a cassette with a constitutive promoter, elongation factor-1 alpha (EF-1α), driving the expression of TPO. To monitor the expression of TPO, the GFP reporter gene was linked downstream to TPO with the use of the E2A fragment, a self-cleaving peptide (Fig. 1a, Additional file 2: Figure S1A). The complete cassette was flanked on each side with 600-bp homology arms to facilitate its integration into the safe-harbor AAVS1 locus, as described previously [25]. To establish the TPO knock-in cell lines, H1 hESCs were transfected with CRISPR plasmids including 717-pEF1-Cas9-wpre-polyA, S479-pU6-AAVS1b, and pD-AAVS1 (HA600)-EF1α-TPO-GFP which express spCas9, sgRNA, and the donor template, respectively [25]. After transfection, GFP^high^ cells were sorted as single cells and then plated for further culture. Two colonies, from those with stable expression of GFP, were selected and expanded for subsequent analyses (designated TPO-KI-1 and TPO-KI-2, respectively) (Fig. 1b, Additional file 2: Figure S1B). The successful insertion of transgene TPO-GFP was initially confirmed through analysis of PCR amplification products of the TPO and GFP genes (Fig. 1c, Additional file 2: Figure S1C). Expression of TPO was further confirmed through real-time PCR and western blotting (Fig. 1d, e). Most importantly, around 195.7 ± 26.75 pg/ml or 202.5 ± 27.64 pg/ml TPO was detected in the supernatant of TPO-KI-1 or TPO-KI-2 cell cultures, respectively (Fig. 1f). To further test whether transgene expression of TPO exerted any effect on hESC pluripotency, the expression of pluripotency makers and teratoma formation capacity were further determined with TPO-KI-1 and TPO-KI-2 cells. A significant difference was not found in the expression of *OCT-4* (also known as *POU5F1*), *SOX2*, and *NANOG*, at either the mRNA or the protein levels (Fig. 1g, Additional file 2: Figure S1D, E). Furthermore, cells with the TPO transgene still maintained the capability to form teratomas (Fig. 1h, Additional file 2: Figure S1F). Thus, using the knock-in strategy at the safe-harbor AAVS1 locus, we established two cell lines with stable TPO expression, allowing us to further determine the effects of autonomous expression of TPO on hematopoietic differentiation and platelet generation.

### TPO knock-in enhances hematopoietic differentiation of hESCs

**Fig. 2:**
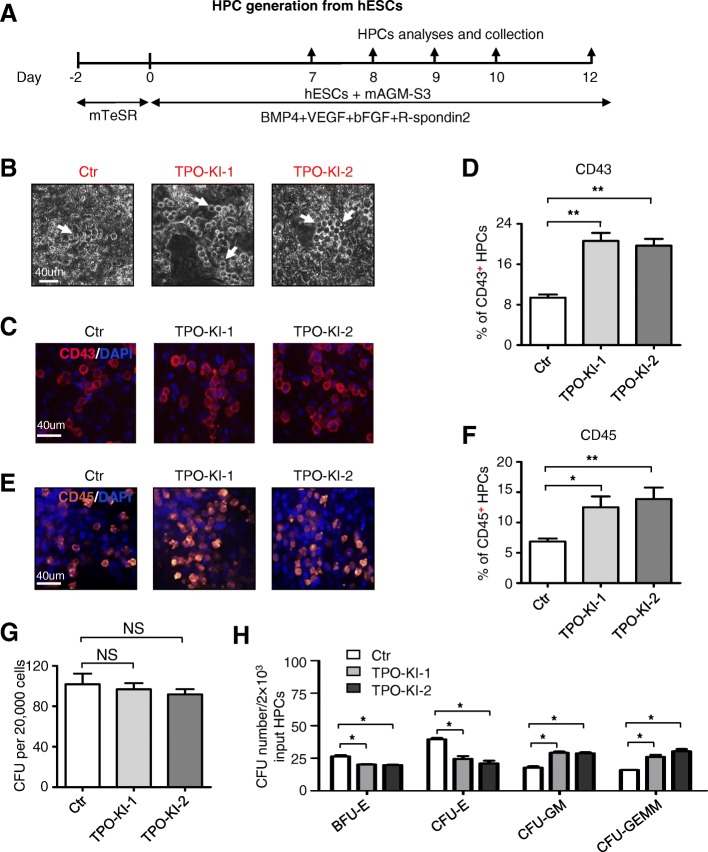
TPO knock-in enhances hematopoietic differentiation of hESCs. **a** Schematic of hematopoietic differentiation from hESCs to HPCs by coculturing with mAGM-S3 stromal cells. **b** Phase-contrast images of derived HPCs from Ctr or TPO-KI-1/-2 H1 hESCs at day 10 of differentiation. Typical cobblestone-like HPCs marked with white arrow. Scale bar = 40 μm. **c** Representative immunofluorescence images displaying generation of CD43^+^ HPCs from Ctr or TPO-KI-1/-2 H1 hESCs at day 7 of differentiation. Scale bar = 40 μm. **d** Flow cytometry analysis showing percentage of CD43^+^ HPCs in Ctr or TPO-KI-1/-2 H1 hESCs at day 7. Data shown as mean ± SEM (*n* = 3). **P < 0.01. **e** Representative immunofluorescence images of CD45^+^ HPCs generated from Ctr or TPO-KI-1/-2 H1 hESCs at day 10. Scale bar = 40 μm. **f** Flow cytometry analysis showing percentage of CD45^+^ HPCs in Ctr or TPO-KI-1/-2 H1 hESCs at day 10. Data shown as mean ± SEM (*n* = 3). *P < 0.05; **P < 0.01. **g**, **h** Hematopoietic colony-forming potential of CD43^+^ HPCs generated from Ctr or TPO-KI-1/-2 H1 hESCs. Total CFU number (**g**) or individual BFU-E, CFU-E, CFU-GM, or CFU-GEMM number (**h**) in Ctr or TPO-KI-1/-2 H1 hESCs calculated. Data shown as mean ± SEM (*n* = 3). *P < 0.05; NS, not significant. bFGF basic fibroblast growth factor, BFU-E burst-forming unit erythroid, BMP4 bone morphogenetic protein 4, CFU colony-forming unit, CFU-E colony-forming unit erythrocyte, CFU-GEMM colony-forming unit granulocyte/erythroid/macrophage/megakaryocyte, CFU-GM colony-forming unit granulocyte/macrophage, Ctr control, DAPI 4′,6-diamidino-2-phenylindole, hESC human embryonic stem cell, HPC hematopoietic progenitor cell, TPO thrombopoietin, VEGF vascular endothelial growth factor

To determine the ability of TPO knock-in hESCs to undergo hematopoietic differentiation, we used a previously described AGM-S3 coculture system [23] (Fig. 2a). Cobblestone-like hematopoietic progenitor cells (HPCs) arose from both control cells and TPO knock-in cells (Fig. 2b). Interestingly, more CD43^+^ HPCs were generated from TPO-KI-1 and TPO-KI-2 cells than control cells without TPO knock-in after a 9-day differentiation (WT vs TPO-KI-1 9.39% ± 0.61% vs 20.63% ± 1.59%, *P* < 0.01; WT vs TPO-KI-2 9.39% ± 0.61% vs 19.69% ± 1.34%, *P* < 0.01) (Fig. 2c, d, Additional file 3: Figure S2A). After further differentiation, significantly more CD45^+^ hematopoietic cells were derived from TPO-KI-1 and TPO-KI-2 cells (WT vs TPO-KI-1 6.25% ± 0.58% vs 12.52% ± 1.80%, *P* < 0.05; WT vs TPO-KI-2 6.25% ± 0.58% vs 13.87% ± 1.91%, *P* < 0.01) (Fig. 2e, f, Additional file 3: Figure S2B). The lineage-specific potential of HPCs was further determined using colony-formation unit (CFU) assay after a 12-day differentiation. Different types of colonies, including BFU-E (burst-forming unit erythroid), CFU-E (colony-forming unit erythrocyte), CFU-GM (colony-forming unit granulocyte/macrophage), and CFU-GEMM (colony-forming unit granulocyte/erythroid/macrophage/megakaryocyte), arose from the plated cells (Additional file 3: Figure S2C). No significant difference was found in the colony numbers after 20,000 cells were plated for each well (Fig. 2g). Interestingly, fewer CFU-E (WT vs TPO-KI-1 40.0 ± 1.1% vs 25.0% ± 2.2%, *P* < 0.05; WT vs TPO-KI-2 40.0% ± 1.1% vs 21% ± 2.1%, *P* < 0.05) and BFU-E (WT vs TPO-KI-1 26.0% ± 1.0% vs 20.0% ± 0.4%, *P* < 0.05; WT vs TPO-KI-2 26.0% ± 1.0% vs 19.0% ± 0.4%, *P* < 0.05) colonies were derived with TPO-KI-1 and TPO-KI-2 cells (Fig. 2h). In contrast, more CFU-GM and CFU-GEMM colonies were derived from cells with TPO knock-in. These results indicate that TPO knock-in not only enhances HPC generation but also causes bias of the differentiation potential of generated HPCs.

### TPO knock-in augments megakaryocytic differentiation

**Fig. 3:**
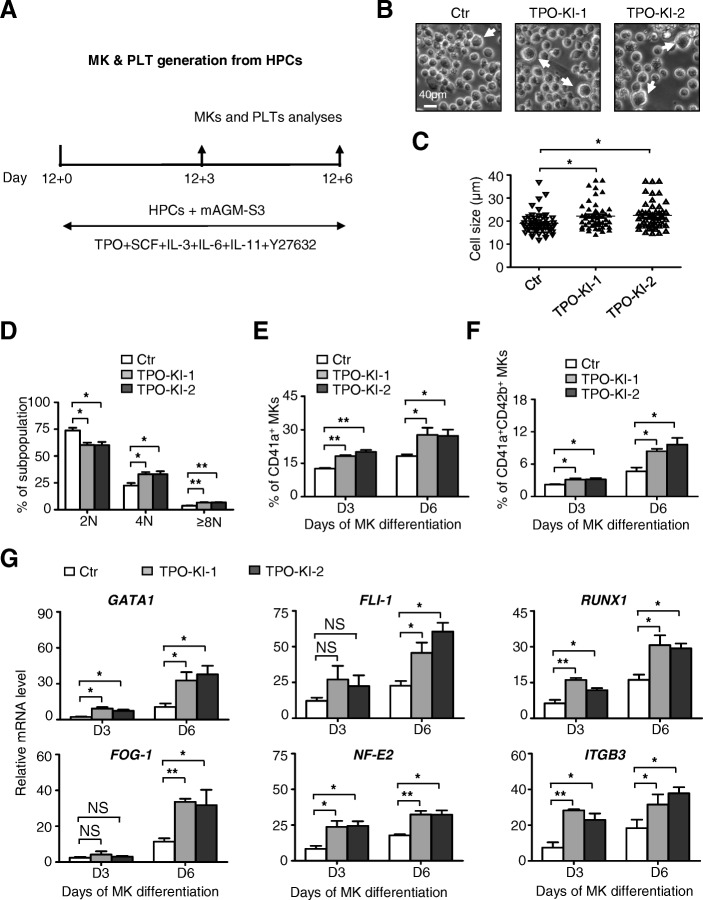
TPO knock-in augments megakaryocytic differentiation. **a** Schematic of megakaryocytic differentiation from HPCs to MKs and PLTs using mAGM-S3 stromal cell coculture. **b** Representative cell morphology at day 6 of megakaryocytic differentiation from Ctr or TPO-KI-1/-2 H1 hESCs. Scale bar = 40 μm. Large cells indicated by white arrows. **c** Distribution of sizes of megakaryocytes formed at day 6 in (**a**) as measured with microscopy. Data shown as mean ± SEM (*n* = 3). *P < 0.05. **d** Ploidy distribution of megakaryocytes analyzed by staining cellular DNA with propidium iodide (PI). Data shown as mean ± SEM (n = 3). *P < 0.05; **P < 0.01. **e, f** Flow cytometer analysis for percentage of CD41a^+^ (**e**) or CD41a^+^CD42b^+^ (**f**) megakaryocytes at day 3 or day 6 of MK differentiation, respectively. Data shown as mean ± SEM (*n* = 3). *P < 0.05; **P < 0.01. **g** qRT-PCR analysis of megakaryocytic-associated markers (*GATA1*, *FLI-1*, *RUNX1*, *FOG-1*, *NF-E2*, *ITGB3*) in Ctr or TPO-KI-1/-2 H1 hESCs for indicated times. *ACTIN* used as internal control. All values normalized to level (=1) of mRNA in Ctr at day 0 of MK differentiation. Data shown as mean ± SEM (*n* = 3). *P < 0.05; **P < 0.01; NS, not significant. Ctr control, HPC hematopoietic progenitor cell, IL interleukin, MK megakaryocyte, PLT platelet, TPO thrombopoietin

Our purpose with autonomous expression of TPO in hESCs is to generate a greater number of megakaryocytes (MKs) and platelets but at a reduced cost. Thus, we further analyzed the megakaryocytic differentiation potential of TPO knock-in HPCs. To induce megakaryocytic differentiation, we harvested HPCs at day 12 of differentiation in the coculture system and then induced them to form megakaryocytes using the step-wise strategy as described previously [23] (Fig. 3a, Additional file 4: Figure S3A). During differentiation, cells with larger sizes began to emerge around day 3 (D3) and then peaked around day 6 (D6) (Fig. 3b). At day 6, a significantly greater quantity of cells with larger size were generated from TPO-KI-1 and TPO-KI-2 cells than with the control cells (Fig. 3c). Furthermore, we observed more MKs with a 4 N or higher level of polyploidy differentiated from TPO-KI-1 and TPO-KI-2 cells than with the control cells (Fig. 3d). In keeping with these results, we found fewer diploid MKs were generated from TPO knock-in cells. Thin-section electron micrography showed that the ultrastructure was very similar between different groups of cells, which contained granules, a typical demarcation membrane system (DMS), and lobulated nuclei (Additional file 4: Figure S3B). We also measured the expression of MK-associated genes, including surface markers such as CD41a^+^, CD42b^+^, and *ITGB3*, and transcription factors such as *GATA1*, *FLI-1*, *FOG-1*, *RUNX1*, and *NF-E2*. In fact, significantly more CD41a^+^ or CD41a^+^CD42b^+^ MKs were produced from TPO-KI-1 and TPO-KI-2 cells (Fig. 3e, f). Furthermore, expression of the aforementioned transcription factors was significantly greater in cells with TPO knock-in (Fig. 3g). In contrast, although a slightly higher number of MKs were derived at day 3 (D3) of differentiation, no difference in cell number was seen at day 6 of differentiation (Additional file 4: Figure S3C). These results demonstrate that TPO knock-in augments the megakaryocytic differentiation potential of hESCs, thus likely facilitating potential large-scale generation of platelets from hESCs.

### More platelets were generated from TPO knock-in cells

**Fig. 4:**
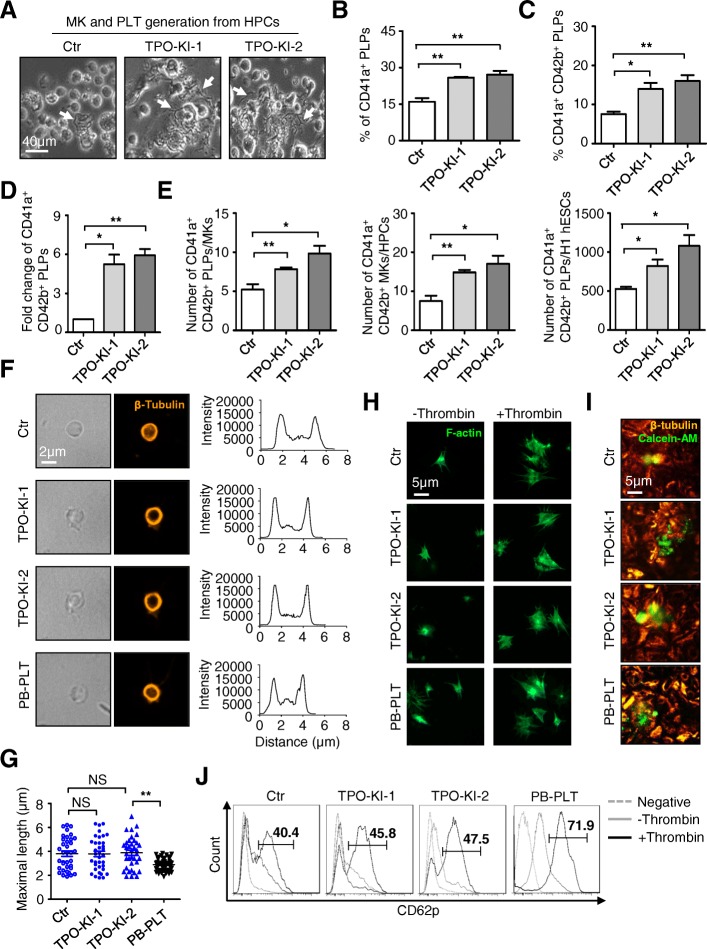
More functional platelets generated from TPO knock-in cells. **a** Representative morphology of proplatelets derived from megakaryocytes in Ctr or TPO-KI-1/-2 H1 hESC groups. Scale bar = 40 μm. **b, c** Flow cytometry analysis showing percentage of CD41a^+^ (**b**) or CD41a^+^CD42b^+^ (**c**) platelets in Ctr or TPO-KI-1/-2 H1 hESC groups, respectively. Data shown as mean ± SEM (*n* = 3). *P < 0.05; **P < 0.01. **d** Fold change of CD41a^+^CD42b^+^ platelets in Ctr or TPO-KI-1/-2 H1 hESC groups, respectively. Data shown as mean ± SEM (*n* = 3). *P < 0.05; **P < 0.01. **e** Comparative analyses of CD41a^+^CD42b^+^ PLTs per MKs or seeded H1 hESCs, MKs per HPCs. Data shown as mean ± SEM (*n* = 3). *P < 0.05; **P < 0.01. **f** Phase-contrast images (left panel) and fluorescence images (right panel) of PB-PLTs or cultured platelets in Ctr or TPO-KI-1/-2 H1 hESC groups. Platelets stained for β-tubulin (microtubule cytoskeleton, orange). Representative line functions for single platelet shown by distance (μm) and intensity (inset). Scale bar = 2 μm. **g** Distribution of maximal particle diameter of PB-PLT or cultured platelets, quantified by immunofluorescence micrographs. Sizes of platelets shown as mean ± SEM (*n* = 3). **P < 0.01; NS, not significant. **h** Immunofluorescence images of platelets bound to immobilized fibrinogen with F-actin filament in absence (left panel) or presence (right panel) of 1 U/ml thrombin. Scale bar = 5 μm. **i** Aggregates of a mixture of 2 × 10^5^ Calcein-AM (red)-labeled blood or cultured platelets and 2 × 10^7^ blood platelets. Red, β-tubulin staining of both populations. Scale bar = 5 μm. **j** Representative flow cytometry analysis showing percentage of P-selectin (CD62P)-positive events in gated CD41a^+^ platelets in indicated groups. Blood platelets (right panel) used as positive controls. Ctr control, hESC human embryonic stem cell, HPC hematopoietic progenitor cell, MK megakaryocyte, PB-PLT platelet from peripheral blood, PLT platelet, TPO thrombopoietin, PLP platelet-like particle

We recently reported a two-step strategy for deriving functionally intact platelets from hESCs [23]. This system was used to test whether the MKs generated from TPO knock-in cells were also able to produce more functional platelets. As expected, more proplatelets, followed by the release of genuine platelets, were observed for both TPO-KI-1 and TPO-KI-2 cells (Fig. 4a). Quantitative analysis with flow cytometry and calculation as reported previously [26] further confirmed the increase in numbers of platelets produced from TPO-KI-1 and TPO-KI-2 cells (Fig. 4b–e, Additional file 5: Figure S4A). A typical discoid shape and a smooth contour were observed in platelets generated from hESCs. These structures were very similar to those of platelets from the peripheral blood (PB-PLT) (Fig. 4f). On average, the proplatelets generated from hESCs were larger than those from PB. This observation is consistent with our previous result that, under static conditions, larger-size platelets were generated from hESCs than platelets from peripheral blood [24]. No difference was observed between cells with or without TPO knock-in (Fig. 4g). Furthermore, platelets contained a normal distribution of the open canalicular system (OCS), while α- and dense granules and mitochondria were observed in different groups of cells (Additional file 5: Figure S4B). We also determined the function of platelets generated from TPO-KI-1 and TPO-KI-2 cells using assays as described previously [24]. When incubated with thrombin, PLPs generated from TPO knock-in cells were able to extend lamellipodia, as demonstrated by F-actin staining (Fig. 4h). The PLPs derived from TPO-KI-1 and TPO-KI-2 cells could also interact with one another and formed large aggregates after agonist stimulation (Fig. 4i). We also measured the functional integrity of platelets generated from TPO-KI-1 and TPO-KI-2 cells using CD62P staining. As expected, CD62P^+^ platelets were observed for different groups of cells (Fig. 4j). Taken together, these analyses confirmed that higher quantity of platelets can be generated from TPO knock-in cells. These platelets exhibit the same morphological, ultrastructural, and functional characteristics as those from blood platelets.

### TPO knock-in partially replaces extrinsic TPO

**Fig. 5:**
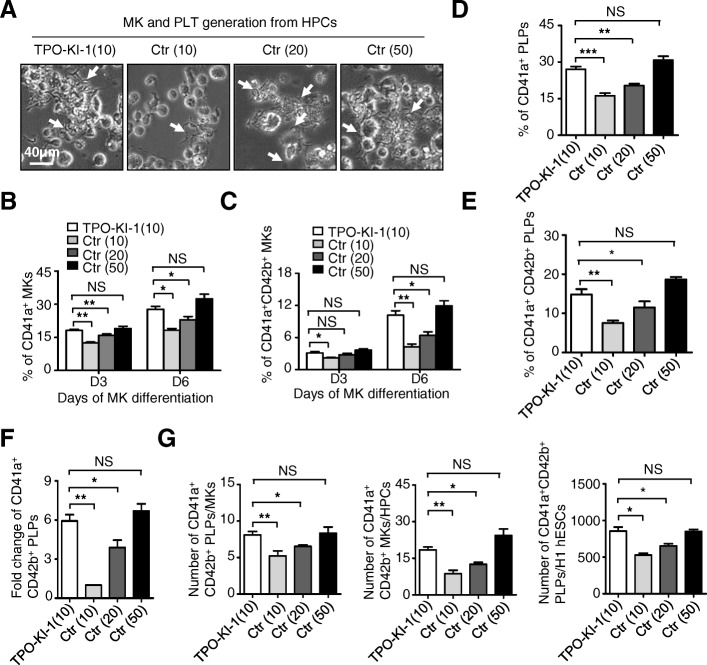
TPO knock-in partially replaces extrinsic TPO. **a** Representative morphology of proplatelets (white arrows) among indicated groups at day 6 (D6) during MK differentiation. Scale bar = 40 μm. **b, c** Flow cytometry analysis showing percentage of CD41a^+^ (**b**) or CD41a^+^CD42b^+^ (**c**) MKs in indicated groups at day 3 (D3) and D6, respectively. Data shown as mean ± SEM (*n* = 3). *P < 0.05; **P < 0.01; NS, not significant. **d, e** Flow cytometer analysis for percentage of CD41a^+^ (**d**) or CD41a^+^ CD42b^+^ (**e**) platelets in indicated groups, respectively. Data shown as mean ± SEM (*n* = 3). *P < 0.05; **P < 0.01; NS, not significant. **f** Fold change of CD41a^+^CD42b^+^ PLPs in indicated groups. Data shown as mean ± SEM (*n* = 3). *P < 0.05; **P < 0.01; NS, not significant. **g** Comparative analyses of CD41a^+^CD42b^+^ PLTs per MKs or seeded H1 hESCs, MKs per HPCs, at day 6 of megakaryocytic differentiation among four groups. Data shown as mean ± SEM (*n* = 3). *P < 0.05; **P < 0.01; NS, not significant. Ctr control, hESC human embryonic stem cell, HPC hematopoietic progenitor cell, MK megakaryocyte, PLT platelet, TPO thrombopoietin, PLP platelet-like particles

We next tested whether the TPO intrinsically produced is capable of replacing the exogenous source. Initially, we sought to generate platelets from the TPO-KI-1 and TPO-KI-2 cells using the same culture condition as described earlier, albeit in the absence of any exogenous TPO. However, in the absence of exogenous TPO, very minimal megakaryotic differentiation was detected and no platelet generation was observed. Thus, autonomous TPO generated from knock-in alone was insufficient to initiate megakaryotic differentiation and thrombopoiesis. We therefore tested whether the knock-in could partially replace exogenous TPO. To test this, we included low concentrations of TPO (10 ng/ml) with TPO-KI-1 and TPO-KI-2 cells. Meanwhile, Ctr cells (GFP knock-in H1 hESCs) were differentiated in the presence of different concentrations of exogenous TPO (i.e., 10 ng/ml, 20 ng/ml, and 50 ng/ml) and were used as controls (Fig. 5a). We assessed the generation of CD41a^+^ and CD41a^+^CD42b^+^ MKs at day 3 (D3) and day 6 (D6), respectively. Consistently, we observed more proplatelets and significantly higher percentages of both the CD41^+^ (TPO-KI-1 vs Ctr (10) 27.7% ± 1.3% vs 18.2% ± 0.8%, *P* < 0.05) and CD41^+^CD42^+^ (TPO-KI-1 vs Ctr (10) 10.2% ± 0.8% vs 4.2% ± 0.5%, *P* < 0.01) MKs from TPO knock-in cells when compared to Ctr cells with the same concentrations of exogenous TPO (10 ng/ml) at D6 (Fig. 5b, c). Interestingly, the TPO knock-in cells also exhibited greater efficiency to generate both CD41a^+^ and CD41^+^CD42^+^ MKs under 10 ng/ml than wild-type cells with 20 ng/ml TPO supplementation (CD41a^+^ MKs, TPO-KI-1 vs Ctrl (20) 27.7% ± 1.3% vs 22.9% ± 1.5%, *P* < 0.05; CD41^+^CD42^+^ MKs, TPO-KI-1 vs Ctrl (20) 10.2% ± 0.8% vs 6.4% ± 0.7%, *P* < 0.05). In fact, the differentiation efficiency is comparable to that of wild-type cells with 50 ng/ml TPO (CD41a^+^ MKs, TPO-KI-1 vs Ctrl (50) 27.70% ± 1.3% vs 32.4% ± 2.1%, NS *P* = 0.39; CD41^+^CD42^+^ MKs, TPO-KI-1 vs Ctrl (50) 10.2% ± 0.8% vs 11.9% ± 1.0%, NS *P* = 0.91) (Fig. 5b, c). Measurements of MK-associated gene expression further confirmed the results from flow cytometry (Additional file 6: Figure S5A). We also assessed platelet generation with different concentrations of exogenous TPO (i.e., 10 ng/ml, 20 ng/ml, and 50 ng/ml). The efficiency of CD41a^+^ or CD41^+^CD42^+^ platelet generation from TPO knock-in cells with 10 ng/ml TPO was comparable to that from wild-type cells with 50 ng/ml TPO (Fig. 5d–f, Additional file 6: Figure S5B) (CD41a^+^ platelets, TPO-KI-1 vs Ctrl (50) 27.0% ± 1.2% vs 30.8% ± 1.6%, NS *P* = 0.11; CD41^+^CD42^+^ platelets, TPO-KI-1 vs Ctrl (50) 14.8% ± 1.4% vs 18.6% ± 0.7%, NS *P* = 0.14). To further quantify the replacement effect of TPO-KI-1 cells on platelet generation, we determined the number of platelets generated per seeded hESC or megakaryocyte, or megakaryocyte generation per HPC, as reported previously [24]. The platelet yields for the TPO-KI-1 cells with 10 ng/ml TPO were comparable to those for the control cells with 50 ng/ml TPO and were much higher than those for the control cells with 10 ng/ml or 20 ng/ml TPO (Fig. 5g) at D6. Thus, TPO knock-in partially replaces the exogenous source of TPO in platelet generation from hESCs. These results also imply that the direct knock-in of various essential cytokines into hESCs might reduce the cost of large-scale platelet generation from hESCs in the future.

## Discussion

To reduce the cost and thereby generate platelets from hESCs on a large scale for future transfusion purposes, we have tested the feasibility of directly knocking-in TPO in hESCs to replace the need for an exogenous source of TPO for platelet generation. By using CRISPR/Cas9-mediated gene recombination, we have successfully established TPO knock-in hESCs with reliable expression of TPO. More importantly, we found that TPO knock-in augments early hematopoiesis, megakaryopoiesis, and thrombopoiesis. The stable autonomous expression of TPO resulting from knock-in partially replaces the exogenous TPO source during platelet generation from hESCs. The development of CRISPR/Cas9 and other genome editing technologies has greatly enabled gene corrections and disease modeling [27–29]. However, to our knowledge, applications of direct knock-in of cytokines to the induction of hESC differentiation have not been reported. Our results therefore provide the first proof-of-concept study for such strategies. Thus, it may be ultimately possible to apply multiple cytokine knock-ins for future experimentation, which may allow for large-scale generation of platelets from hESCs in cytokine-free conditions and at significantly reduced costs.

Currently, platelet transfusion is the first-line treatment for trauma and numerous hematological malignancies. Unfortunately, all platelets come from donors, which may limit the available supplies during times of emergency, indicating the need for large-scale production of platelets from stem cells in vitro for patient transfusion, which has yet to be accomplished [3, 30]. Although hESCs may serve as the potential source for clinical and therapeutic supplies, achieving the necessary quality and achieving the necessary quantity of platelets from hESCs are the two main challenges remaining. We and others have recently demonstrated that integration of biomechanical force and biochemical signals improves both the functional integrity and the efficiency of platelet production [24, 31]. Although clinical trials in patients are still lacking, most functional tests conducted in vitro or in animal models have shown that the platelets generated in vitro are comparable with those isolated from peripheral blood [1, 32]. In contrast, cost-effective generation of clinically scalable platelet production remains a major challenge. Due to the ability of unlimited expansion, hESCs can be an ideal cell source for platelet production. Feng et al. [2] reported a method to generate HLA-ABC^negative^ universal platelets from β2M^KO^ iPSCs in large scale under serum/feeder-free conditions with bioreactors. This represents an important advancement in the effort to tackle the issue of limited platelet quantity [2]. When compared to iPSCs regarding the donor of the cell source, the ethics of ESCs might be a problem for clinical applications. Thus, the same strategy will be further tested on hiPSCs for future clinical purposes. To avoid potential variations caused by feeder cells, Liu et al. [33] recently reported successful generation of megakaryocytes, but not platelets, using Food and Drug Administration (FDA)-approved pharmacological reagents. However, for all of the reported methods, supplementation with high concentrations of cytokines, such as TPO, SCF, and IL-3, is required, making it costly to perform even a single transfusion (i.e., 200–300 billion platelets) when multiple transfusions are often required. Our attempt with TPO knock-in results in partial replacement of the extrinsic source of TPO and improved derivation of HPCs, megakaryocytes, and platelets, indicating that the strategy might prove promising for cost reduction of future large-scale platelet generation. Similarly, we envision that the same knock-in strategy might also be applied to the generation of other functional cell types from hPSCs.

Interestingly, TPO knock-in alone fails to induce differentiation in the absence of TPO. Furthermore, we observed silencing of GFP/TPO expression at the late stage of megakaryocytic differentiation and platelet generation (data not shown). Thus, silencing of TPO expression might partially explain why knock-in alone cannot completely replace extrinsic TPO. As such, a better understanding of the control at the level of expression, activity, and stability of target cytokines requires further exploration. Future experimental efforts will focus on optimization of locus targeting and multiple knock-ins of various cytokines to further improve platelet generation.

## Conclusions

Overall, our data demonstrate that hematopoietic differentiation, megakaryocytic differentiation, and platelet generation are benefited by autonomous production of TPO in hESCs via CRISPR/Cas9-mediated knock-in. This cytokine knock-in strategy could be cost-effective for future large-scale generation of platelets in translational medicine.

## Additional files

Additional file 1: Additional Information. Additional Figure Legends, Additional Experimental Procedures, Additional Tables S1–S3, and Additional References. (DOCX 44 kb)

Additional file 2: **Figure S1.** Identification of TPO-KI H1 hESCs by flow cytometer analysis of GFP^+^ population, agarose gel electrophoresis of amplified PCR products, qRT-PCR and western blotting analysis of pluripotency markers, or imaging analysis of teratoma. (PDF 201 kb)

Additional file 3: **Figure S2.** TPO-KI accelerates early hematopoiesis of hESCs. HPCs for indicated times confirmed and quantified by flow cytometry, cell counting, or hematopoietic colony-forming analysis. (PDF 266 kb)

Additional file 4: **Figure S3.** TPO-KI promotes hESC megakaryocytic differentiation. MK generation or total cell number confirmed and quantified by thin-section electron micrographs or cell counting, respectively. (PDF 219 kb)

Additional file 5: **Figure S4.** TPO-KI augments platelet production. Platelet microparticles or ultrastructure gated by flow cytometry or identified by thin-section electron micrographs, respectively. (PDF 109 kb)

Additional file 6: **Figure S5.** TPO-KI partially replaces extrinsic TPO in platelet production. qRT-PCR analysis of megakaryocytic-associated markers and flow cytometer analysis for percentage of CD41a^+^, CD42b^+^ platelet microparticles performed. (PDF 85 kb)

## Abbreviations

BFU-E: Burst-forming unit erythroid
CFU-E: Colony-forming unit erythrocyte
CFU-GEMM: Colony-forming unit granulocyte/erythroid/macrophage/megakaryocyte
CFU-GM: Colony-forming unit granulocyte/macrophage
DMS: Demarcation membrane system
HPC: Hematopoietic progenitor cell
MK: Megakaryocyte
PLP: Platelet-like particle
PLT: Platelet
TPO: Thrombopoietin
