# Supplementary material for: Thrombopoietin knock-in augments platelet generation from human embryonic stem cells
Source: Stem Cell Res Ther. 2018 Jul 17;9:194. doi: 10.1186/s13287-018-0926-x (PMC6050740; doi:10.1186/s13287-018-0926-x)
Supplement: Supplementary file 1 — Additional Information. Additional Figure Legends, Additional Experimental Procedures, Additional Tables S1–S3, and Additional References. (DOCX 44 kb) [file 13287_2018_926_MOESM1_ESM.docx]

**Additional Information**

**Thrombopoietin Knock-In Augments Platelet Generation from Human Pluripotent Stem Cells**

Leisheng Zhang^1,2^, Cuicui Liu^1,2^, Hongtao Wang^1,2^, Dan Wu^1,2^, Pei Su^1,2^, Mengge Wang^1,2^, Jiaojiao Guo^3^, Shixuan Zhao^1,2^, Shuxu Dong^1,2^, Wen Zhou^3^, Cameron Arakaki^4^, Xiaobing Zhang^1,2,4^, Jiaxi Zhou^1,2*^

**Additional Figure Legends for Addition Fig S1-5;**

**Additional Experimental Procedures;**

**Additional Tables 1-3;**

**Additional References.**

**Figure S1. Relative to Figure 1.** **Identification of TPO-KI H1 hESCs**

**(A)** Schematic illustration of homologous recombination of the TPO gene into the AAVS1 locus on chromosome 19. **(B)** Flow cytometer analysis for the percentage of GFP^+^ cells in Ctr or TPO-KI (-1, -2) H1 hESCs. **(C)** Identification of Ctr or TPO-KI (-1, -2) H1 hESCs by using agarose gel electrophoresis of the amplified PCR products with designed primers listed in Additional Table 1. (**D**) qRT-PCR analysis of pluripotency markers (*POU5F1, SOX2, NANOG*) in Ctr or TPO-KI (-1, -2) H1 hESCs in mTeSR. All values are normalized to the level (=1) of mRNA in Ctr. Data are shown as mean ± SEM (n=3). NS, not significant. (**E**) Western blotting analysis confirms the expression of pluripotency markers (*OCT4, SOX2, NANOG*) in Ctr or TPO-KI (-1, -2) H1 hESCs in mTeSR. α-Tubulin is used as a loading control. **(F)** Phase contrast images (top panel) and fluorescence images (bottom panel) of teratomas formed by Ctr or TPO-KI (-1, -2) H1 hESCs. Scale bar=1cm.

**Figure S2. Relative to Figure 2.** **TPO-KI accelerates early hematopoiesis of hESCs**

**(A)** and **(B)** Dynamic analyses of the percentage of CD43^+^ (A) or CD45^+^ (B) HPCs for indicated times by flow cytometry. **(C)** Fold of changes of total HPCs at day 12 of hematopoietic differentiation was analyzed. **(D)** Hematopoietic colony-forming potential of CD43^+^ HPCs detached from Ctr or TPO-KI (-1, -2) H1 hESCs. Representative morphologies of BFU-E, CFU-E, CFU-GM and CFU-GEMM are shown. Scale bar, 1cm.

**Figure S3. Relative to Figure 3.** **TPO-KI promotes hESC megakaryocytic differentiation**

**(A)** Schematic illustration of megakaryocytic differentiation of hESC-derived HPCs. HPCs at day 12 of hematopoietic differentiation were detached and cocultured with mAGM-S3 stromal cells for megakaryocytic differentiation. Large megakaryocytes and proplatelet are pointed by black and white arrows, respectively. Scale bar, 20μm. **(B)** Thin-section electron micrographs of culture-derived megakaryocytes in Ctr or TPO-KI (-1, -2) group. Scale bar, 10μm. **(C)** Total cell numbers in the Ctr or TPO-KI (-1, -2) group were counted with cell counter. Results are shown as mean ± SEM (n = 3). *, *P*<0.05; NS, not significant. Abbreviations: DMS, demarcation membrane system; G, granules; N, nuclei.

**Figure S4. Relative to Figure 4.** **TPO-KI augments platelets production**

**(A)** Representative flow cytometer analysis for the percentage of CD41a^+^, CD42b^+^ platelet microparticles at day 6 of megakaryocytic differentiation from Ctr or TPO-KI (-1, -2) H1 hESC-derived HPCs using mAGM-S3 stromal cell co-culture with 10ng/ml TPO addition. **(B)** Thin-section electron micrographs of culture-derived platelet in Ctr or TPO-KI (-1, -2) group. PB-PLT was used as a positive control. Scale bar, 10μm.

**Figure S5. Relative to Figure 5.** **TPO-KI partially replaces extrinsic TPO in platelets production**

**(A)** qRT-PCR analysis of megakaryocytic-associated markers (*GATA1, FLI-1, RUNX1, FOG-1, NF-E2, ITGB3*) in Ctr or TPO-KI-1 H1 hESCs for indicated times. *ACTIN* was used as an internal control. All values are normalized to the level (=1) of mRNA in Ctr at day 0 of MK differentiation. Data are shown as mean ± SEM (n=3). *, *P*<0.05; **, *P*<0.01; NS, not significant. **(B)** Representative flow cytometer analysis for the percentage of CD41a^+^, CD42b^+^ platelet microparticles at day 6 of megakaryocytic differentiation from TPO-KI-1 or Ctr hESC-derived HPCs using mAGM-S3 stromal cell co-culture with indicated concentration of TPO addition.

**Additional Experimental Procedures**

**Construction of the Plasmids**

For Cas9 and sgRNA Plasmids Construction, PCR products were produced using KAPA HiFi polymerase (KAPA Biosystems) and purified using a GeneJET Gel Extraction Kit (Thermo Fisher Scientific). The linear PCR products were then assembled into plasmids in a DNA assembly reaction (20 μL), on ice, according to the manufacturer’s instructions. The reaction contained NEBuilder HiFi DNA Assembly Master Mix (10 μL), equal ratios o PCR products (0.2–0.5 pmols), and deionized water. The ligation reaction was briefly vortexed and centrifuged prior to incubation at 50 °C for 5–30 min. NEB 5-alpha Competent E. coli cells were then transformed with the assembled DNA products and plated on ampicillin-treated agar plates.

To construct pJET donor plasmids (TPO-E2A-GFP and GFP), all of the fragments (left HA, EF1α-TPO-E2A-GFP-wpre or EF1α-GFP-wpre, right HA) were amplified by PCR using KAPA HiFi polymerase (KAPA Biosystems) and purified using a GeneJET Gel Extraction Kit (Thermo Fisher Scientific). To clone donor plasmids harboring sgRNA recognition sites, the sgRNA target sequence together with a PAM (NGG), were included in both the forward and the reverse primers. A ligation reaction (20 uL) was performed, on ice, according to the manufacturer’s instructions, containing 2X Reaction Buffer (10 uL), pJET1.2/blunt Cloning Vector (50 ng/μL) (1 uL), T4 DNA Ligase (1 uL), purified PCR product (0.15 pmol), and nuclease-free water (remaining volume). The ligation reaction was then briefly vortexed and centrifuged prior to incubation at room temperature (22 °C) for 5–30 min. NEB 5-alpha Competent E. coli cells were then transformed with the ligation product and plated on ampicillin-treated agar plates.

**Western Blotting**

Protein of cells are prepared and detected as we previously reported[[1](#_ENREF_1)]. Briefly, 5×10^6^ cells are collected and lysed with 100μl laemmli sample buffer (BioRad). Proteins are separated by using SDS-PAGE and transferred onto nitrocellulose filter membrane (WhatMan). Then the membrane was blocked by 5% nonfat milk for 1hr at room temperature. Different concentrations of various primary antibodies were listed in Additional Table S1. HRP-conjugated secondary antibodies (Sigma-Aldrich) were used for incubations (1:5000). The blots were developed with the Super-Signal West Pico Chemiluminescent Substrate system (Pierce). The antibodies were listed in Additional Table S2.

**Ploidy Analysis of Megakaryocytes**

To measure the DNA content of megakaryocytes, ploidy analysis was performed as we previously reported with several modifications[[2](#_ENREF_2),[3](#_ENREF_3)]. Briefly, 5×10^5^ cells at day 6 of megakaryocyte differentiation were collected and labeled with APC-CD41a antibody. Then, after 30 minutes’ fixation with 4% paraformaldehyde (Solarbio), the cells were permeabilized with 0.2% Triton-100 (Sigma-Aldrich) for 20 minutes. Next, the cells were treated with 100μg/ml RNase (Solarbio) for 30min and labeled with 40mg/ml propidium iodide (Sigma-Aldrich) for 15 minutes. The ploidy analysis of megakaryocyte was performed by gating CD41a^+^ population using Canto Ⅱ (BD) flow cytometry.

**Platelets Enrichment and Purification**

The platelets in the supernatant were collected by centrifugation at 300g for 10 minutes. Next, the platelets were resuspended in 2 ml CGS buffer with 1μM PGE1 (Sigma), and spun at a BSA density [gradient](javascript:void(0);) [centrifugation](javascript:void(0);) of 80g for 10 minutes as we previously did. The upper layers containing purified platelets were centrifugated for 10 minutes 800g for 10 minutes. The purified platelets were resuspended in CGS buffer and maintained at room temperature for further morphological and functional assay.

**Immunofluorescence of Platelets**

Platelets isolated from peripheral blood or derived by coculture were plated onto 35mm confocal dishes (Corning) or 15 mm coverslips coated with poly-L-lysine (100μg/ml) at 37℃ for 1 hr. Then, the platelets were fixed, permeabilized and blocked as we previously did with several modifications. For characterization assay, platelets were labeled with mouse anti-β1-tubulin antibody, then incubated with 594-conjugated goat anti-mouse IgG (Bio). The fluorescent images of platelets were recorded under a confocal microscopy (LSM710) and analyzed with the velocity software (velocity software 4.0). The diameter of platelets was measured by Nano Measurer software (Nano Measurer 1.2) based on β1-tubulin staining.

**Aggregation and Adhesion Test of Platelets**

To test the aggregation potential of platelets, 2×10^5^ purified culture-derived platelets or peripheral blood platelets were labeled with Calcein-AM (Invitrogen), and mixed with 2×10^7^ peripheral blood platelets. Next, the mixed platelets were centrifugated onto fibrinogen-coated coverslips in 24-well plates at 80g for 5 minutes. Then, washed with 1×PBS and incubated the coverslips on a shaker with 300 μl of culture medium containing Thrombin (1U/ml), fibrinogen (300μg/ml) and ADP (20μM) at 37℃ for 20 minutes. Then, the aggregates were labeled with mouse anti-β1-tubulin and 594-conjugated goat anti-mouse IgG (Bio). Images of platelets were visualized under a confocal microscopy (LSM710).

To test the adhesion potential of platelets, 1×10^6^ purified culture-derived platelets or peripheral blood platelets were plated onto fibrinogen (1000μg/ml)-coated coverslips in the presence of absence of thrombin (2U/ml) at 37℃ for 1 hr. Then, washed with 1×PBS and labeled the platelets with fluorescein isothiocyanate 488-conjugated phalloidin. Images of platelets were visualized under a confocal microscopy (LSM710).

**Additional Tables**

**Table S1. Primers used in this study. Related to Fig. 1,3 and Fig. S1, S5.**

Real-time PCR primer sequences. Related to Fig. 1, 3 and Fig. S1, S5.

| Gene | Forward Primer | Reverse Primer |
| --- | --- | --- |
| *ACTIN* | CTCTTCCAGCCTTCCTTCCT | AGCACTGTGTGTTGGCGTACAG |
| *POU5F1* | CTTGAATCCCGAATGGAAAGGG | GTGTATATCCCAGGGTGATCCTC |
| *SOX2* | GCCGAGTGGAAACTTTTGTCG | GGCAGCGTGTACTTATCCTTCT |
| *NANOG* | TTTGTGGGCCTGAAGAAAACT | AGGGCTGTCCTGAATAAGCAG |
| *GATA1* | CACTGAGCTTGCCACATCC | ATGGAGCCTCTGGGGATTA |
| *RUNX1* | TCTTCACAAACCCACCGCAA | CTGCCGATGTCTTCGAGGTTC |
| *FLI-1* | GGCCTGAACAGTAGAGGCG | CACCGGAGACTCCCTGGAT |
| *FOG-1* | CGTGCTTCGAGTGCGAGAT | CGCCTCTACTGTTCAGGCC |
| *NF-E2* | CGGCGCAGCGAATATGTAGA | CCGACGTTCATCCCGACTC |
| *ITGB3* | GTGACCTGAAGGAGAATCTGC | CCGGAGTGCAATCCTCTGG |
| *GFP* | CATCCTGGTCGAGCTGGACG | AGCACTGCACGCCGTAGGTC |
| *TPO* | AACTGCAAGGCTAACGCTGT | GACATGGGAGTCACGAAGCA |
| *GAPDH* | GAGGTGTGAGTGGGATGGTGG | GCCTGCTTCACCACCTTCTTG |

Primer sequences for GFP or TPO knock-in identification. Related to Fig. 1 and Fig. S1.

| Primer Name | Primer Sequence |
| --- | --- |
| F1 | CTTGCTCTGCTGTGTTGC |
| F2 | TTCCTTCTCGGCGCTGCACC |
| F3 | GGCTTCTGAAGTGGCAGCAG |
| F4 | GCTGCTGGACAGGGGCTCGG |
| F5 | GCTCGCCTGTGTTGCCACCT |
| F9 | GGCACCTCGATTAGTTCTCG |
| F10 | CATCCTGGTCGAGCTGGACG |
| R1 | GGTGTAGGCAAAGGGTGAAC |
| R2 | ACAAGCAGGAGGAGCCGGGC |
| R3 | AGGGAAGCGGGACCCTGCTC |
| R4 | GTGGACGAGGAAGGGGGACA |
| R5 | AGCACTGCACGCCGTAGGTC |
| R6 | CACCGAGCTGCAAGAACTCT |
| R7 | CCGCGCCACCTTCTCTAGGC |
| R8 | CGACTACTGCACTTATATAC |
| ACTIN-F | CTCTTCCAGCCTTCCTTCCT |
| ACTIN-R | AGCACTGTGTGTTGGCGTACAG |
| pJET1.2-F | CGACT CACTATAGGGAGAGCGGC |
| pJET1.2-R | AAGAA CATCGATTTTCCATGGCAG |
| U6-F | GGGCAGGAAGAGGGCCTAT |
| EF1-F | GGCTCCGGTGCCCGT |
| wpre-R | GCCCAAAGGGAGATC |

**Table S2. Antibodies used in this study. Related to Fig.1-5 and Fig. S2-4.**

Antibodies for flow cytometry and immunofluorescence. Related to Fig. 2-5 and Fig. S2, S4.

| Antibody | Cat. NO. | Source |
| --- | --- | --- |
| Anti-CD41a-APC | 555751 | BD Pharmigen |
| Anti-CD42b-PE | 555473 | BD Pharmigen |
| Anti-CD43-PE | 560199 | BD Pharmigen |
| Anti-CD43-APC | 560199 | BD Pharmigen |
| Anti-CD45-PE | 560975 | BD Pharmigen |
| Anti-CD62P-PE | 555524 | BD Pharmigen |
| 488 donkey anti-rabbit IgG | R37118 | Invitrogen |
| 594 donkey anti-mouse IgG | R37115 | Invitrogen |
| Anti-β-Tubulin I antibody | SAB4200715 | Sigma-aldrich |
| Phalloidin-iFluor 488 Conjugate | AAT-23115 | AAT Bioquest |

Antibodies for western-blotting assay. Related to Fig. 1-2, Fig. S1.

(I: immunofluorescence; W: western blotting)

| Name | Company | Catalog | Host | Dilution |
| --- | --- | --- | --- | --- |
| TPO | Sigma | SAB1400278 | Rabbit | 1:1000(W) |
| GFP | ProteinTech | 66002-1-Ig | Mouse | 1:2000(W) |
| OCT3/4 | Santa Cruz | SC-9081 | Rabbit | 1:200(I)/1:1000(W) |
| SOX2 | Millipore | AB5603 | Rabbit | 1:200(I)/1:500(W) |
| NANOG | Cell Signaling | 3580 | Rabbit | 1:200(I)/1:300(W) |
| α-Tubulin | Abcam | Ab11304 | Mouse | 1:10000(W) |
| GAPDH | ProteinTech | 10494-1-AP | Rabbit | 1:2000(W) |

**Table S3. Chemical compounds. Related to Fig.3-5 and Fig. S3-5.**

| Reagent | Cat. NO. | Source | Concentration/(ng/ml) |
| --- | --- | --- | --- |
| hTPO | 300-18 | PEPROTECH | 0, 10, 20, 50 |
| hSCF | 300-07 | PEPROTECH | 20 |
| hIL-3 | 200-03 | PEPROTECH | 10 |
| hIL-6 | 200-06 | PEPROTECH | 10 |
| hIL-9 | 200-09 | PEPROTECH | 10 |
| hIL-11 | 200-11 | PEPROTECH | 10 |
| Y27632 | S1049 | SELLECK | 10 |
| hR-spondin2 | 120-43 | PEPROTECH | 20 |

**Additional References**

1. Wu Q, L Zhang, P Su, X Lei, X Liu, H Wang, L Lu, Y Bai, T Xiong, D Li, Z Zhu, E Duan, E Jiang, S Feng, M Han, Y Xu, F Wang and J Zhou. (2015). MSX2 mediates entry of human pluripotent stem cells into mesendoderm by simultaneously suppressing SOX2 and activating NODAL signaling. Cell Res; 25:1314-32.

2. Yang Y, C Liu, X Lei, H Wang, P Su, Y Ru, X Ruan, E Duan, S Feng, M Han, Y Xu, L Shi, E Jiang and J Zhou. (2016). Integrated Biophysical and Biochemical Signals Augment Megakaryopoiesis and Thrombopoiesis in a Three-Dimensional Rotary Culture System. Stem Cells Transl Med; 5:175-85.

3. Wang H, C Liu, X Liu, M Wang, D Wu, J Gao, P Su, T Nakahata, W Zhou, Y Xu, L Shi, F Ma and J Zhou. (2018). MEIS1 Regulates Hemogenic Endothelial Generation, Megakaryopoiesis, and Thrombopoiesis in Human Pluripotent Stem Cells by Targeting TAL1 and FLI1. Stem Cell Reports; 10:447-460.
