# Supplementary figures and images for: Thrombopoietin knock-in augments platelet generation from human embryonic stem cells
Source: Stem Cell Res Ther. 2018 Jul 17;9:194. doi: 10.1186/s13287-018-0926-x (PMC6050740; doi:10.1186/s13287-018-0926-x)

Additional Fig. S1

**A**

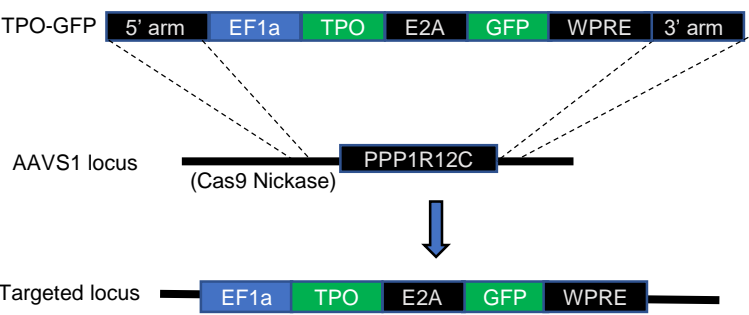

**C**

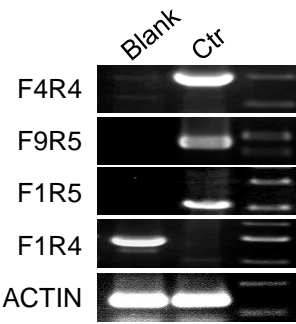

**B**

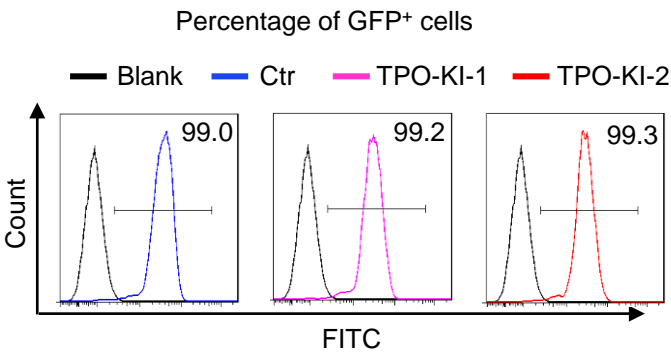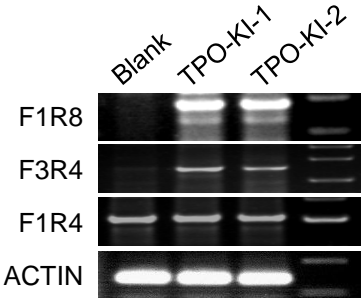

**D**

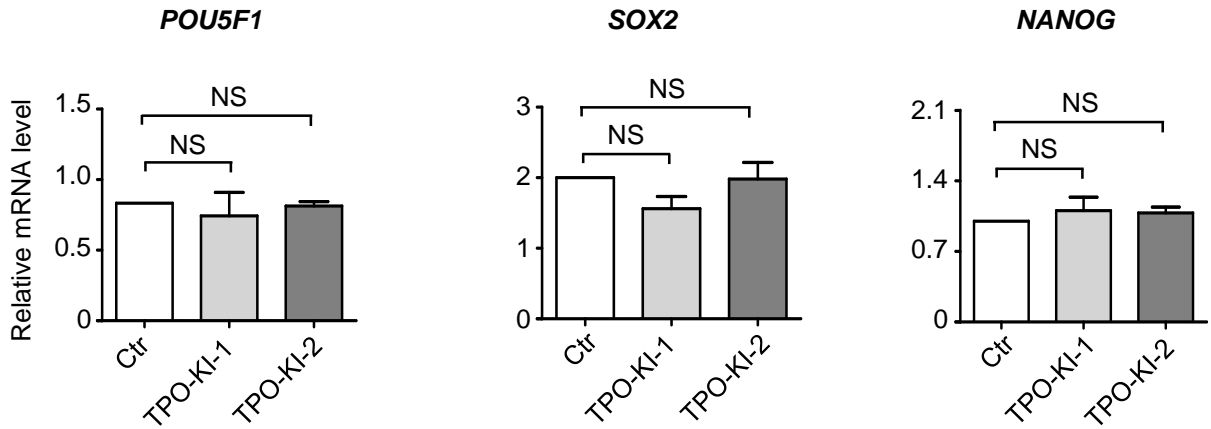

**E**

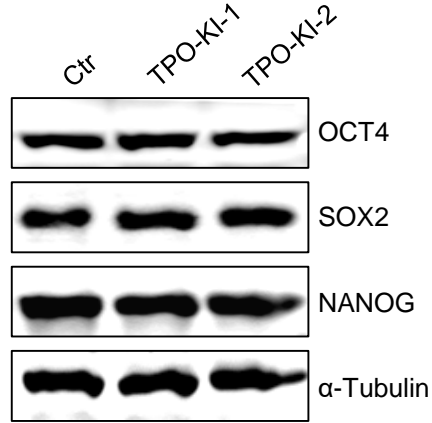

**F**

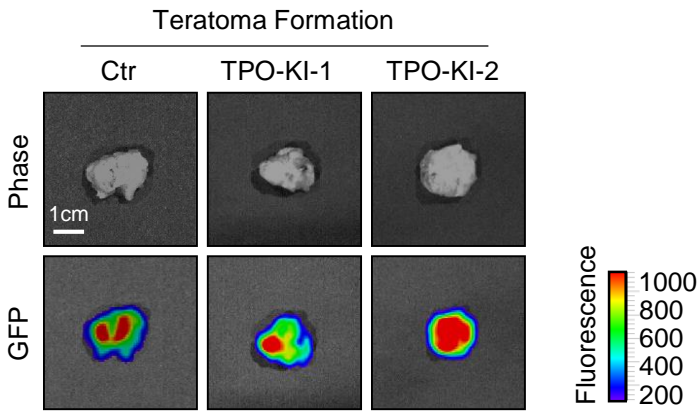

Supplement: Supplementary file 2 — Figure S1. Identification of TPO-KI H1 hESCs by flow cytometer analysis of GFP+ population, agarose gel electrophoresis of amplified PCR products, qRT-PCR and western blotting analysis of pluripotency markers, or imaging analysis of teratoma. (PDF 201 kb) [file 13287_2018_926_MOESM2_ESM.pdf]

Additional Fig. S2

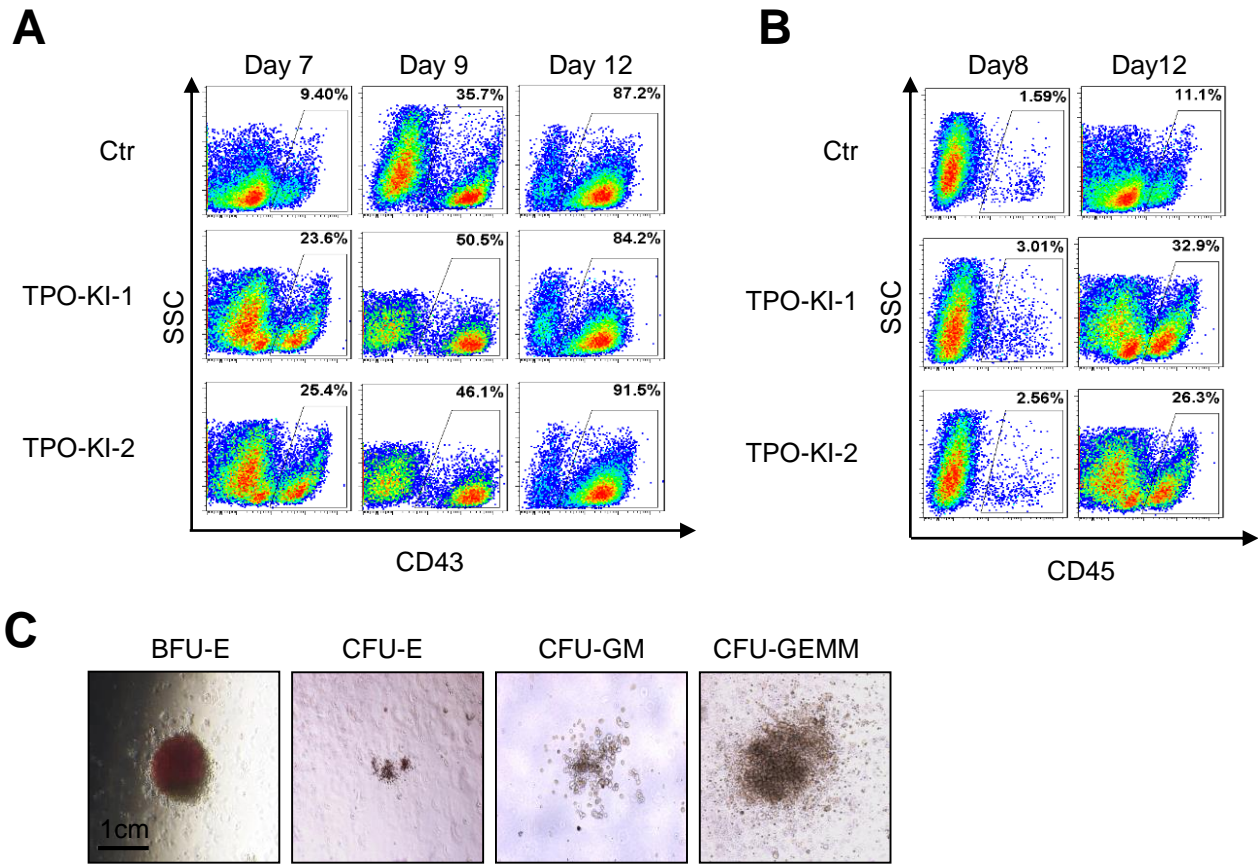

Supplement: Supplementary file 3 — Figure S2. TPO-KI accelerates early hematopoiesis of hESCs. HPCs for indicated times confirmed and quantified by flow cytometry, cell counting, or hematopoietic colony-forming analysis. (PDF 266 kb) [file 13287_2018_926_MOESM3_ESM.pdf]

# Additional Fig. S3

**A**

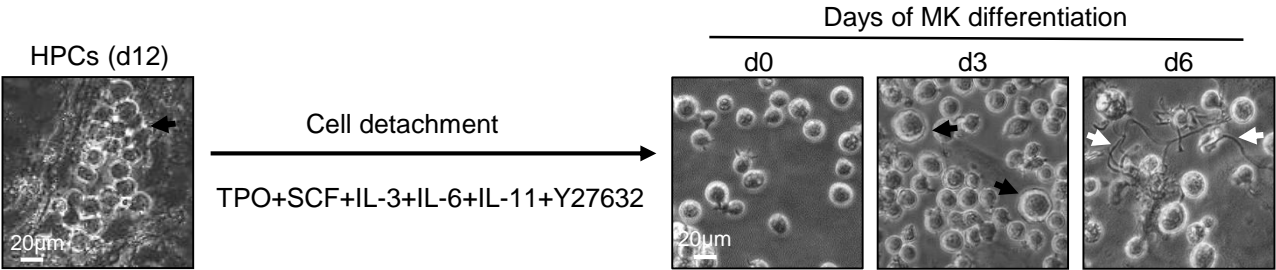

**B**

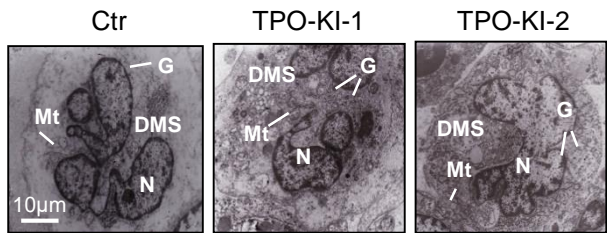

**C**

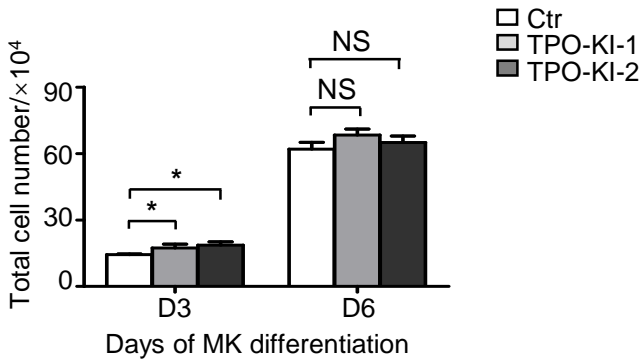

Supplement: Supplementary file 4 — Figure S3. TPO-KI promotes hESC megakaryocytic differentiation. MK generation or total cell number confirmed and quantified by thin-section electron micrographs or cell counting, respectively. (PDF 219 kb) [file 13287_2018_926_MOESM4_ESM.pdf]

Additional Fig. S4

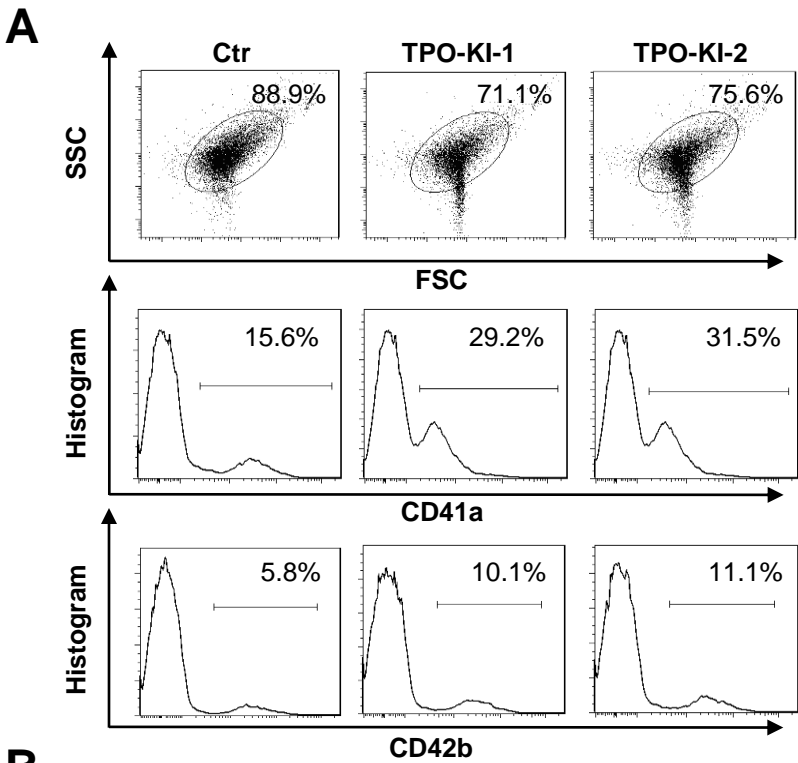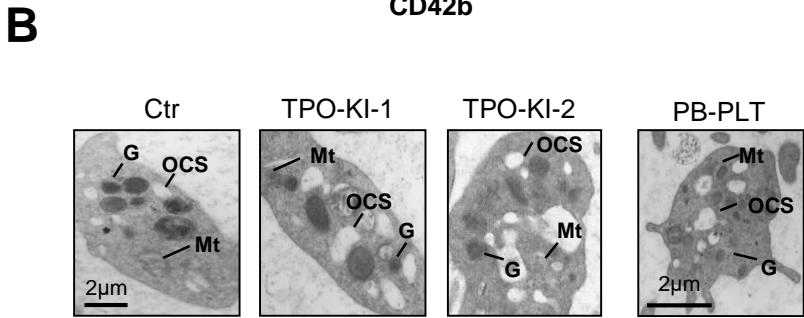

Supplement: Supplementary file 5 — Figure S4. TPO-KI augments platelet production. Platelet microparticles or ultrastructure gated by flow cytometry or identified by thin-section electron micrographs, respectively. (PDF 109 kb) [file 13287_2018_926_MOESM5_ESM.pdf]

# Additional Fig. S5

**A**

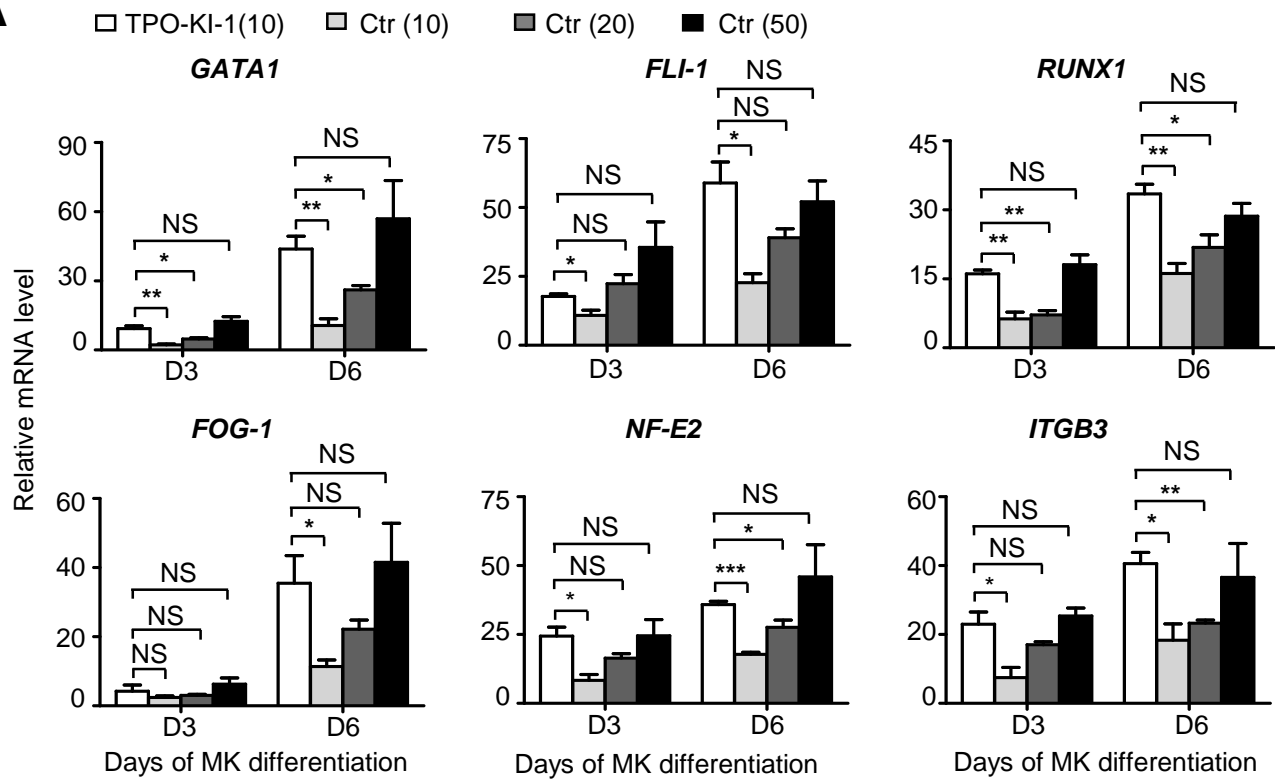

**B**

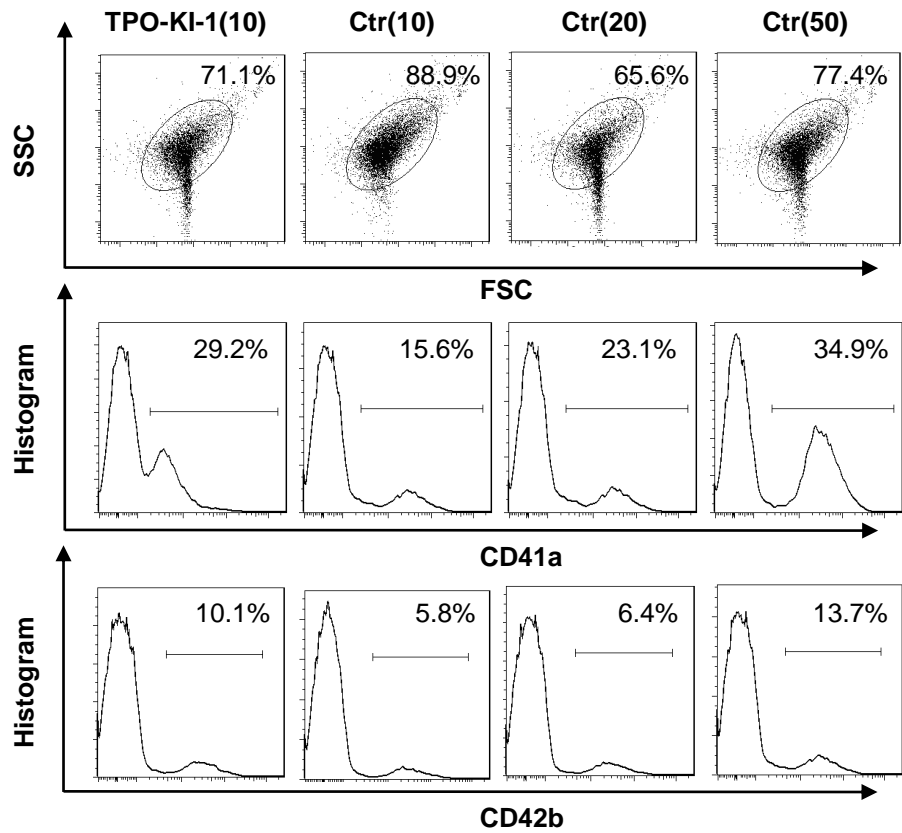

Supplement: Supplementary file 6 — Figure S5. TPO-KI partially replaces extrinsic TPO in platelet production. qRT-PCR analysis of megakaryocytic-associated markers and flow cytometer analysis for percentage of CD41a+, CD42b+ platelet microparticles performed. (PDF 85 kb) [file 13287_2018_926_MOESM6_ESM.pdf]
